# Establishing human responsibility and accountability at early stages of the lifecycle for AI-based defence systems

**DOI:** 10.1007/s10676-025-09862-1

**Published:** 2025-10-06

**Authors:** Ariel Conn, Ingvild Bode

**Affiliations:** 1 The University of Colorado, Boulder, USA; 2 Center for War Studies, University of Southern Denmark, Odense, Denmark

**Keywords:** Human control, AI, Weapon systems, Military systems, Autonomy, Responsibility, Accountability, AI lifecycle

## Abstract

The use of AI technologies in weapons systems has triggered a decade-long international debate, especially with regard to human control, responsibility, and accountability around autonomous and intelligent systems (AIS) in defence. However, most of these ethical and legal discussions have revolved primarily around the point of use of a hypothetical AIS, and in doing so, one critical component still remains under-appreciated: human decision-making across the full timeline of the AIS lifecycle. When discussions around human involvement start at the point at which a hypothetical AIS has taken some undesirable action, they typically prompt the question: “what happens next?” This approach primarily concerns the technology at the time of use and may be appropriate for conventional weapons systems, for which humans have clear lines of control and therefore accountability at the time of use. However, this is not precisely the case for AIS. Rather than focusing first on the system in its comparatively most autonomous state, it is more helpful to consider when, along the lifecycle, humans have more clear, direct control over the system (e.g. through research, design, testing, or procurement) and how, at those earlier times, human decision-makers can take steps to decrease the likelihood that an AIS will perform ‘inappropriately’ or take incorrect actions. In this paper, we therefore argue that addressing many arising concerns requires a shift in how and when participants of the international debate on AI in the military domain think about, talk about, and plan for human involvement across the full lifecycle of AIS in defence. This shift includes a willingness to hold human decision-makers accountable, even if their roles occurred at much earlier stages of the lifecycle. Of course, this raises another question: “How?” We close by formulating a number of recommendations, including the adoption of the IEEE-SA Lifecycle Framework, the consideration of policy knots, and the adoption of Human Readiness Levels.

Since the mid-2010s, questions around integrating autonomy and AI into the military domain have been the subject of international debate. Initially, this debate happened exclusively within the Convention on Certain Conventional Weapons (CCW) with a focus on so-called lethal autonomous weapon systems (AWS) that, once activated, “select and apply force to targets without human intervention” (ICRC, 2021, p. 1). Since 2022, the debate has spread across various forums such as the Responsible AI in the Military Domain (REAIM) Summits and Regional Consultations and the United Nations (UN) General Assembly First Committee. Simultaneously, the focus expanded to cover AI in the military domain more broadly across areas such as decision support, logistics, and cyber (Grand-Clément, 2023). To speak to this broader range of uses of autonomous and AI technologies in the military domain, we refer to autonomous and intelligent systems (AIS) in defence throughout this paper (IEEE Research Group on Issues of AI and Autonomy in Defence Systems, 2024, p. 11).

One of the most controversial issues in the debate has long been the legal, moral, and ethical implications of a system ‘acting’ without human control. Over time, maintaining adequate human oversight, control, or involvement has emerged as a shared principle across governance initiatives and discussions (Bode, 2025). This principle underscores the essential function of human control in maintaining human accountability and responsibility in use-of-force decisions, particularly to ensure compliance with international law (e.g. UN-CCW, 2019).

Within policy debates, practical questions around implementing human responsibility and accountability have increasingly acknowledged that many human decision-makers were involved in designing, testing, and approving the system across its lifecycle. Despite this, international and regulatory discussions around AIS in defence continue to privilege the use stage in two ways. First, the primary, most discussed concern is what to do if using the system has resulted in some sort of problematic action and who will be held accountable in such cases. Second, earlier lifecycle stages of an AIS—those that occur before decisions are made to deploy the system—are typically only referenced in passing rather than in detail. Even one of the most detailed publications to spell out the lifecycle of human decision-making, the UN Institute for Disarmament Research’s (UNIDIR) Iceberg infographic, begins at the stage of political decision-making with regard to potentially using a particular, developed system (2020).

We argue that discussing AIS in this way risks missing human decision-making across the full timeline of the AIS lifecycle as a key factor in maintaining human control. Although political and military leaders must remain responsible and accountable for decisions over the use of force, their decisions will be heavily influenced by their understanding of how the system will work, how reliable it is, and how predictable the outcomes of use will be. This type of knowledge will necessarily draw from documentation and demonstrations regarding the earlier stages of research, development, testing, and acquisition and procurement.

In making this argument, we build on recent work by the IEEE SA Research Group on Issues of AI and Autonomy in Defence Systems, of which we both were co-authors. The IEEE SA Research Group has produced the first detailed framework to break up the stages of the AIS lifecycle and looks at these stages, including the earliest, through the lens of human decision-making (2024). This work revealed that there continue to be significant gaps in understanding between different groups involved across the lifecycle. Legal and political experts may be familiar with international discussions around AIS, but less familiar with how the technology works or with the existing standards and norms that inform decision-making at early stages of systems engineering. Meanwhile, engineers and technical researchers may understand the technology, but they are less familiar with how humans will use and interact with the system at later stages, and, in particular, they lack familiarity with international humanitarian law (IHL) and other relevant legal frameworks. The significance of these gaps of understanding for establishing responsibility and accountability have been recognized for a while (Bloch et al., 2022). Even as some efforts have been made to bridge these gaps with respect to AI more generally, military systems are typically excluded from ethical and normative guidance provided here, though there are a few notable exceptions (Gillespie, 2019; See et al., 2019; Walker-Munro & Assaad, 2023). To ensure responsibility and accountability across the full AIS lifecycle, more work needs to be done by people from a variety of fields at earlier stages for each system that is developed. This paper therefore looks more closely at components of the international discussions that contribute to the gap in understanding and considers additional frameworks that can help bridge the gap.

The remainder of the paper proceeds by first, exploring human decision-making across the full timeline in greater detail at a conceptual level. Second, we contrast conventional weapon systems with those integrating autonomous and AI technologies to consider why conversations need to shift. Third, we summarise key concerns arising with regard to human decision-makers across the full lifecycle and offer solutions to better address responsibility and accountability, drawing primarily on insights offered by the IEEE SA Framework, along with other models, such as Human Readiness Levels (HRLs).

For the purpose of this paper, we understand human responsibility in a legal sense, covering both establishing whether an internationally wrongful act (state responsibility) or a war crime (individual criminal responsibility) has occurred (Bo et al., 2022, p. 2). In turn, accountability refers to “the act of ensuring that relevant officials or institutions are answerable for actions and that there is recourse in situations where obligations are not met” (Dorsey & Bonacquisti, 2017, p. 27). Yet, we have to recognise that responsibility and accountability are often used interchangeably in the international debate. This could be owing to the fact that the (rather) precise distinctions found in the English language do not appear to translate well into other languages such as French, German, or Mandarin.

## The role of humans along the full timeline of the AIS lifecycle

When discussions remain focused on AIS at the time of use, neither the responsible human decision-makers nor the full timeline that incorporates all relevant human decision-makers can be properly taken into account. This leads to an insufficient line of reasoning. Consider, for example, a common concern that often arises during such discussions: “What happens if an AIS misidentifies a target and uses force against a civilian? Who will be held responsible and accountable?” The phrasing of the questions in this way naturally draws attention *to the AIS at the time of use*. The first question centers on the system, while the word choice suggests that the AIS is a decision-making entity. The framing and word choice serve to remove human decision-making from the outcomes resulting from using the AIS and suggest the absence of human involvement in the most important moment of decision-making. As a result, the second question, which belatedly brings the human back into the scenario, leads to the commonly identified ethical, legal, and moral challenges.

Reframing the questions we ask in discussions about AIS can begin to shift the focus back to the diverse groups of human decision-makers. For example, the questions above could be reframed to say, “what happens if a human decision-maker permits or oversees the deployment of an AIS that then results in the use of force against civilians? Looking back across the full lifecycle of human decision-making, how were responsibility and accountability transferred throughout the development and testing of the AIS, and who are the humans who were responsible and accountable at different stages?” Asking questions in this way may not solve some of the trickiest problems in the debate, such as establishing an ‘appropriate’ requisite quality of human control, addressing all responsibility and accountability concerns, or addressing other legal challenges (Bode, 2023; Bruun et al., 2023; Crootof, 2022). Yet this reframing can better enable policymakers, military leadership, and legal and ethical experts to look at the full lifecycle of the system and thereby consider if and how international law might need to be adapted or created to incorporate decision-makers earlier in the lifecycle, along with how to better ensure the appropriate humans remain responsible and accountable.

One reason that the need for human control or oversight poses such difficulties and disagreements is that it is the inverse of so-called ‘full’ autonomy. If autonomy is thought of as a spectrum (see Table 1)—as it often is—then complete human control, in which the system is entirely manual and has no autonomous capabilities at all, would be placed on one side, while ‘full’ autonomy would sit on the opposite side of the spectrum, where humans have no control over the system once activated at all (Bode & Huelss, 2022; Sharkey, 2016). We can find practically no existing weapons systems at the two extreme ends of the spectrum: manual human control and ‘full’ autonomy. Even for a gun, once the bullet leaves the chamber, the human no longer has control. Similarly, ‘fully’ autonomous systems with absolutely no human control or involvement throughout the lifecycle are unlikely to ever be developed, let alone deployed, as such systems would pose unacceptable levels of risk for military organisations structured around control. We can therefore question whether the very idea of ‘full’ autonomy is illusory in and of itself (Jones, 2015, p. 81), and we can consider the spectrum of autonomy outlined in Table 1 as depicting ideal-typical rather than necessarily empirically realistic distinctions. The understanding it provides still serves as background knowledge in the international debate about AWS, in particular (Bode, 2024). For our paper, it is important to acknowledge that most AIS in defence will be found in the middle of this spectrum, exhibiting various forms of human-machine interaction and integrating automated, autonomous, and AI technologies that vary in their complexity.

**Table 1:**
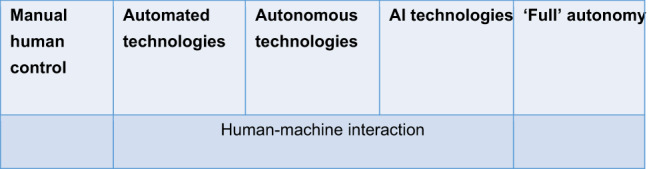
Spectrum from human control to full autonomy. This spectrum could be applied to each stage of the lifecycle. At the earliest stages of the lifecycle, all activities will be under manual human control, but throughout the lifecycle, the levels of autonomy will increase

Considering autonomy as a spectrum, which is essentially a two-dimensional line, also has the drawback of potentially overlooking other important issues. For this paper, the primary component that gets lost is the timeline of human decision-makers. To address this, we first consider some relevant differences between conventional weapons systems and AIS.

## Conventional weapons systems versus AIS in defence

Existing international law, especially international humanitarian law (IHL), was almost entirely developed with respect to conventional weapons systems. These laws were drafted and negotiated assuming that the mechanisms and mechanics of the weapon are predictable and well enough understood so that commanders and operators can be reasonably held responsible for misuse, illegal use, or unintended consequences. For conventional weapons systems, and even some of the early, existing systems integrating automated technologies, such as cruise missiles, this remains true. For these systems, commanders and operators have sufficient understanding of how the system works, enabling them to use the system in the correct context and to reasonably expect and predict specific outcomes as a result of using the system. Such systems can therefore be expected to produce outputs in a predictable manner. This paper assumes that if something does go wrong with a conventional weapons system, then the cause is likely due to:

- human error at the time of use, in which case, the operator or commander will likely be held responsible and accountable;
- human error at earlier stages, in which case the system was flawed to begin with and responsibility and accountability will fall with the developer or procurement officers; or.
- damage to the physical structure of the system, in which case, the type of damage and what caused it will help determine who is to be held responsible and accountable.

In almost all cases, because conventional weapons are predictable, it should be possible to ensure that the correct human(s) will be held responsible and accountable.

However, predictability issues begin to surface even in systems that integrate AI’s predecessor technologies, such as more complex forms of automation. Air defence systems provide a useful illustration. In use by at least 89 militaries around the world (Boulanin & Verbruggen, 2017, p. 37), various types of air defence systems have long integrated automated and autonomous technologies in their targeting functions (Watts & Bode, 2021). More specifically, air defence systems typically function in different modes of human-machine interaction, including versions of automatic mode where the system “can automatically sense and detect targets and fire upon them” (Roff, 2016) without requiring further human input within its pre-programmed parameters (Bode & Watts, 2021, p. 27). In practice, even this integration of automated technologies already limits the quality of human control that human operators can exercise at the point of use. As well-documented failures involving air defence systems that led to the downing of civilian airplanes and incidents of friendly fire illustrate, ways of operating such systems have exhibited unpredictable and problematic outcomes (Bode & Watts, 2021, Chap. 4).

Systems integrating autonomy, especially AI-enabled autonomy, are even more difficult to understand and predict. Common issues that arise with various AI technologies—the black box problem,^1^ bias,^2^ ‘hallucinations,’^3^ adversarial attacks,^4^ etc.—will also likely arise when those AI technologies are added to weapons systems (Blanchard & Bruun, 2024; Holland Michel, 2020; Huelss, 2024; Nadibaidze et al., 2024). Because AI systems do not process information in the same way humans do, it can be challenging to predict how AI will ‘behave’, as well as how and when AI will fail.

Such unpredictability poses major legal concerns. Article 36 of the Geneva Convention mandates weapons testing to ensure predictability, and more recently, the International Committee of the Red Cross (ICRC) has advocated for a prohibition of unpredictable autonomous weapon systems (2021, p. 1). Most militaries also believe it is in their best interest to test weapons systems to ensure the systems will produce outcomes as predicted. Testing and legal reviews can help assure militaries that their weapons will not violate IHL and that the systems will not cause damage to their own members or their allies. Conventional weapons systems have existing protocols and standards for testing and assurance, allowing militaries to deploy them with some quantified measure of assurance that they have and will produce outcomes as predicted.

Additionally, with most conventional systems, humans have some level of control and oversight throughout the entire mission, and in most cases, humans can recall the system if the mission changes. A commonly identified selling point to militaries in favor of AIS is that these systems will be able to function outside of direct human control in areas where communication may be limited, denied, or non-existent (Etzioni & Etzioni, 2017). In those situations, no human would be able to recall the system, change the mission, or stop an attack.

It is expected that any AIS will also be heavily tested for safety and assurance, and verification processes must exist to ensure that it ‘behaves’ predictably. Efforts have already been underway for the past years around clarifying how to conduct Article 36 reviews specifically for AWS (Boutin & Woodcock, 2024; e.g. Chengeta, 2016; Copeland et al., 2023; Walker-Munro & Assaad, 2023). Such work is currently expanded towards providing guidance for legal reviews beyond AWS towards AI-based decision support systems that apply to use-of-force decision-making (Blanchard & Bruun, 2025; Dorsey & Bo, 2025; Klonowska, 2022). As part of this, initiatives such as the UNODA & SIPRI *Handbook on Responsible Innovation in AI for International Peace and Security* as well as Article 36 Legal’s Lawful By Design recognise that such efforts need to entail familiarising technical and industry experts involved in earlier AIS lifecycle stages with the requisite legal and ethical frameworks to ensure responsibility and accountability (Article 36 Legal, 2025; UNODA & SIPRI, 2025).

However, for now, common standards for testing AIS in defence do not exist, and attempts to create regular and consistent processes for testing are still in the very early stages (Bloch et al., 2022). Technologists have struggled to keep up with the development of various standards and testing required to ensure that AI systems in defence meet the requirements of International Humanitarian Law (IHL) and other military needs, given the rapid advancement of AI technologies and their diverse capabilities in recent years. Additionally, as more reports come out regarding the fallibility and dysfunctionality of machine learning-based AI technologies, such as the hallucinations of large language models, the reliability of more advanced autonomous technology is increasingly in question (Raji et al., 2022; Viveros Álvarez, 2024).

Because conventional weapons systems are more straightforward, they are easier to define, making it clearer when a specific body of international law, norm, or standard must apply. With automated, autonomous, and AI technologies being incorporated into nearly every capability, from navigation and obstacle avoidance to the decision to attack a target, the definition of an AIS in defense—or indeed, an autonomous weapons system—continues to be fuzzy and full of limitations (Taddeo & Blanchard, 2022). Most importantly for this paper, responsibility and accountability will be hard to attribute if the use of an AIS in defence causes unintended harm, regardless of whether it fits current definitions. This is why the paper focuses on defining the human roles that influence the development and use of the AIS rather than the particularities of the systems.

## Considering the full lifecycle of AIS

Most laws and regulations that address weapons systems focus on the time of use and the impacts of use, but, as noted above, these laws were developed for conventional weapons systems, which are under human control at the time of use. However, AIS are in their most autonomous states at the time of use. To fully understand responsibility and accountability, one must consider the entire timeline of the lifecycle, as the possibility of human control and oversight increases earlier along this timeline. For AIS, it is necessary to expand the timeframe to include the responsible human decision makers at earlier stages of the system’s lifecycle, including the humans who made decisions regarding first its development and then its deployment.

Such a line of thinking has been expressed, for example, by the idea of policy knots that consider how the interplay of policy, design, and practice unfolds across time in the context of ethically challenging technological development (Jackson et al., 2014; Jones, 2015). A consistent back-and-forth connection between policymakers, technical developers, and users of the system can enable a more profound understanding of issues that arise from each perspective, and it can help lead to the development of more appropriate norms and standards to address these issues. By emphasising this interplay between different actors in the early stages, human agency and decision-making become key components of the technology. Moreover, in addition to bringing the developers and users into policy discussions, the model also highlights the importance of continuing to examine how policy needs might change throughout the lifecycle of an AIS as its capabilities are developed and how its potential use may evolve as needs and contexts may change.

Yet to establish new models for human interaction throughout AIS development, we require a fine-grained understanding of the lifecycle, especially at early stages (Blanchard et al., 2024). The most comprehensive version to date of a lifecycle framework in the military context has been developed by the IEEE SA Research Group on Issues of AI and Autonomy in Defence Systems (2024). This framework identified nine lifecycle stages and five ongoing activities in which human decision makers are most important and influential.

The nine lifecycle stages are:

1. Before AIS development:

- General legal, ethical, and related technical concerns identified and addressed.
- Rationale for military development and use of AIS, formulation of system requirements, and considering the role of researchers.

2) Research and development.
3) Procurement and acquisition.
4) Testing, evaluation, verification, and validation (TEVV).
5) Considering the human: Education, training, and human-system integration.
6) Political and strategic considerations.
7) Operational-level command and control.
8) Tactical employment.
9) Review, reuse, and/or retire.

The five ongoing activities are:

1. Evaluation of legal, ethical, and policy concerns.
2. Responsibility, accountability, and knowledge transfers.
3. Considering the human: training, education, and human-system integration.
4. TEVV, monitoring, hardware system or software updates and interoperability, maintenance.
5. Risk assessments.

The nine lifecycle stages, as listed above, are sufficiently straightforward and self-explanatory that further details about most of the stages will not be provided here, and readers are encouraged to review the full white paper. The ongoing activities are a unique feature compared to most lifecycle frameworks, as they serve as a reminder that, although this is referred to as a ‘cycle’ with what appear to be discrete stages, there are various elements and activities that must occur repeatedly throughout the lifecycle, in a similar fashion to that described by the policy knot model, to ensure that humans remain responsible and accountable—and that the AIS produces outputs as expected. In contrast to conventional systems, for which responsibility and accountability are (and can be) established at later stages, it is important to note that responsible decision makers at any AIS stage listed above could and possibly should be held accountable if it is found that decisions they made negatively affected the outcome of an AIS deployment. That said, to establish humans as responsible and accountable, the most important stages are stage 1 (Before AIS development), stage 5 (considering the human: education, training, and human-system integration), and all five of the ongoing activities.

Stage 1.a. of the IEEE SA Lifecycle Framework highlights the importance of the international discussions referenced in this paper. During these discussions, the groundwork is laid to ensure governments, militaries, weapons developers, and other relevant actors are well-positioned to communicate with each other and to assign and document responsibility and accountability throughout the full AIS lifecycle. As such, a key recommendation of this paper is to more carefully consider language choices when discussing AIS at the time of use, as described in Sect. 1 above. Rather than inadvertently suggesting that an AIS simply has agency, language choices and framing should reflect the human decisions that led to the outcomes of the use of the AIS. This is a simple human activity that, occurring at this early stage, can help ensure assignment and documentation of human responsibility and accountability at later stages.

Stage 1.b. involves making the first fundamental decisions about the creation and application of an AIS, as well as its components. This stage might include members of the military. leadership, representatives of weapons manufacturers, and possibly policymakers, but it might also include AI developers who are working in research or for commercial companies and who may not yet anticipate that the programmes they are developing will be used in weapons systems. At this point, ongoing activities should start, including the potential use of models such as policy knots. Additionally, existing standards, such as the IEEE 7000 Standard Series should be assessed here to ensure these are implemented as appropriate to address potential ethical concerns (IEEE, 2025).

“Considering the human” was thought to be so important by the diverse experts constituting the IEEE-SA Research Group that it is both a discrete stage (stage 5) and an ongoing activity. A component of “considering the human” is ensuring that commanders and operators have sufficient training and information about the system so that they can be reasonably held accountable for it, even if they do not maintain direct control in specific use-of-force situations, as they might with a conventional weapons system. Some questions for people involved to consider include: has the user been sufficiently trained on the user interface? Is the data being presented in a format and with a speed that the user can keep up with? Does the user understand the system’s limitations to prevent unintended misuse? If the answer to these types of questions is ‘no’ and something goes wrong at the point of deployment, then it may not be reasonable to hold the operator or even the commander accountable. In these situations, responsibility and accountability may need to be placed on decision-makers at earlier stages.

One model that could be implemented is the Human Readiness Levels (HRLs) model (Salazar et al., 2020; See et al., 2019). A standard for HRLs was recently adopted by the US Department of Defense to ensure that the human component of any technical system has been considered, designed for, and tested before a system is considered ready to move on to the next stage of the lifecycle (Office of the Under Secretary of Defense for Research and Engineering, 2025). The nine stages of the HRLs were developed to coincide with Technical Readiness Levels (TRLs), which were developed by NASA as part of the Apollo program: “The HRL scale provides organization and program management with a simple rating (ranging from 1 to 9) of the level of maturity of a technology with respect to its readiness for human use. The HRL scale focuses on the degree to which human systems evaluation activities and processes have been completed to demonstrate that a relevant technology or system achieves the level of human readiness needed to meet desired mission objectives” (Human Factors and Ergonomics Society, 2021, p. 3) Initial research suggests that implementing HRLs or similar models improves consideration of the human component throughout the lifecycle (See et al., 2019).

## Conclusion

This paper reviewed the extent to which the international debate about AI in the military domain addresses the roles played by multiple human decision-makers across the AIS lifecycle for maintaining human control and, by association, responsibility and accountability. We found that despite a growing interest in the whole lifecycle approach across the diversifying forums where that debate happens, human decision-makers at the point of use still tend to be privileged in considering and establishing human control. Building on work by the IEEE SA Research Group on AI and Autonomy in Defence Systems, we argued for paying more attention to what human control looks like throughout the entire AIS lifecycle and how responsibility and accountability can be assured throughout.

Part of this effort will require an approach different from that used with conventional means and methods of warfare. Existing laws were written for conventional systems in mind, which have more straightforward premises of human control and tend to focus primarily on the time of use. New or updated legal requirements and regulations may need to be made for AIS in defence, such that responsible human decision-makers from earlier stages of the AIS lifecycle can be identified and held accountable. What is most critical is that militaries and weapons developers need to recognise the significance of choices and decisions made at the earlier stages of the AIS lifecycle. Changing how we discuss AIS concerns can help shift the focus from the technology back to the human. Additionally, practicing a policy knot approach can help bring more people from the policy, development, and use stages together. However, a detailed understanding of the full lifecycle of the AIS is critical to identify who needs to be part of that model.

This leads us to highlight five practical recommendations based on our reading of the IEEE SA Research Group’s efforts. First, responsibility, accountability, and knowledge transfers across all stages can ensure that someone at each stage is responsible and accountable and that relevant knowledge about the system is transferred throughout. Second, issues found with TEVV should start to appear during development stages, but then TEVV should continue repeatedly throughout the entire lifecycle to ensure that old issues are addressed and that any new issues are promptly found before the system does something it should not. Third, human users should receive sufficient training throughout the lifecycle, not just once; this training should cover the system’s capabilities and limitations, including how it can and cannot be used in different contexts. This ensures that humans continue to be legitimate decision-makers rather than merely responding to data that the system generates too quickly. HRLs provide a proven framework for ensuring humans are considered. Fourth, if the use of the system seems to turn human users into button pushers and therefore risks only securing nominal human control (Bode & Nadibaidze, 2024; GCSP, 2024), this needs to be flagged in earlier stages during testing. All of this will help mitigate risks, but risk assessments are still expected to take place routinely throughout the entire lifecycle. Fifth, and most importantly, following this framework, especially in combination with related models, can help ensure that we have clear documentation regarding who is responsible and accountable throughout the full lifecycle of the weapons system.

Addressing these key concerns allows us to move on from asking whether or not human control exists to addressing what human control looks like throughout the entire AIS lifecycle to ensure responsibility and accountability. The lines of work highlighted in this paper offer promising pathways towards adopting and standardising new models and frameworks that enable responsibility and accountability transfers along the timeline.

## Data Availability

No datasets were generated or analysed during the current study.

## Footnotes

1 The black box problem describes the basic challenge of understanding how opaque, deep learning based systems process data and produce outputs (Castelvecchi, 2016).

2 In the context of AI in the military domain, bias can be understood as “the phenomenon of a military AI system being unintentionally inclined towards or against certain individuals or groups of people in a way that is systemic and unfair” (Bruun & Bo, 2025 executive summary).

3 Hallucinations refers to the well-documented tendency of generative AI models to produce factually incorrect outputs.

4 Adversarial attacks of, for example machine vision, target the input data of machine learning systems in either digital and physical ways leading to misclassifications or misidentifications (Elsayed et al., 2018).
